# Facile Preparation of Superhydrophobic PDMS Polymer Films with Good Mechanical Strength Based on a Wear-Resistant and Reusable Template

**DOI:** 10.3390/polym16152165

**Published:** 2024-07-30

**Authors:** Zhi Chen, Shuang Lu, Yumeng Wei, Guojun Zhang, Fenglin Han

**Affiliations:** 1 State Key Laboratory of Precision Manufacturing for Extreme Service Performance, College of Mechanical and Electrical Engineering, Central South University, Changsha 410083, China; 217052@csu.edu.cn (Z.C.); 223711019@csu.edu.cn (S.L.); yumengw@csu.edu.cn (Y.W.); 2 Guangdong Provincial Key Laboratory of Manufacturing Equipment Digitization, Guangdong HUST Industrial Technology Research Institute, Dongguan 523808, China; 18202764498@163.com

**Keywords:** superhydrophobic polymer films, wire electrical discharge machining, wear-resistant and reusable template, mechanical strength

## Abstract

In this paper, a new method involving a wear-resistant and reusable template is proposed for the preparation of high-mechanical-strength superhydrophobic polymer film based on wire electrical discharge machining (WEDM). A solid−liquid-contact-angle simulation model was established to obtain surface-texture types and sizes that may achieve superhydrophobicity. The experimental results from template preparation show that there is good agreement between the simulation and experimental results for the contact angle. The maximum contact angle on the template can reach 155.3° given the appropriate triangular surface texture and WEDM rough machining. Besides, the prepared superhydrophobic template exhibits good wear resistance and reusability. PDMS superhydrophobic polymer films were prepared by the template method, and their properties were tested. The experimental results from the preparation of superhydrophobic polymer films show that the maximum contact angle of the polymer films can be up to 154.8° and that these films have good self-cleaning and anti-icing properties, wear resistance, bending resistance, and ductility.

## 1. Introduction

The term “superhydrophobic surfaces” refers to surfaces with a contact angle greater than 150° and a rolling angle less than 10° [1]. Surface roughness is an important factor that affects dynamic wetting behavior. Within a certain range, the contact angle increases with an increase in roughness and the correct degree of roughness can improve surface hydrophobicity [2,3]. At present, many scholars mainly obtain superhydrophobic surfaces by constructing micro/nano rough structures on the substrate surface or modifying the material surface with low-surface-energy substances. Beyond their superhydrophobic performance, the polymer superhydrophobic surfaces also have excellent characteristics such as transparency, corrosion resistance and higher toughness. These polymer superhydrophobic films are thus widely used in the fields of self-cleaning [4,5,6], anti-icing [7,8], corrosion resistance [9], and oil-water separation [10,11,12].

Through the study of solid-surface wetting theory and the discovery of biological superhydrophobic surfaces in nature, researchers have created many methods for preparing polymer superhydrophobic films, including the template method, the coating method, the etching method, etc. The template method first processes micro/nano structures on the template by a certain processing method. The micro/nano structures are copied from the template to the target surface with low surface energy. Thus, polymer superhydrophobic films are obtained [13,14]. The template method is a commonly used method for preparing superhydrophobic polymer films because of its advantages of easy shape control, simple operation, and suitability for large-scale preparation.

The key to preparing polymer superhydrophobic surfaces using the template method is the preparation of templates, which usually need to have characteristics such as simple preparation, good wear resistance, and reusability. Many scholars have used different templates to prepare polymer superhydrophobic films, including natural and artificially manufactured templates. As for natural templates, insects [15], leek leaves [16], taro leaves [17], soot [18,19,20,21], wood [22,23,24], and protein amyloid fibers [25] have been used as templates to prepare polymer superhydrophobic surfaces. The use of these templates to prepare polymer films could yield films with superhydrophobic properties, with the advantages of simple preparation methods and low cost. However, most natural templates are disposable and not suitable for industrial large-scale production. Additionally, 1800# stainless steel mesh [26] and 1000# sandpaper [27] have been proposed as templates for preparing superhydrophobic fluororubber films. These templates required long preparation times to prepare polymer superhydrophobic templates and required some subsequent processing, such as vulcanization and removal of sand particles. In addition, the chemical-etching method has been proposed to manufacture the microstructure of the template surface; these microstructures would then be replicated onto the polymer surface [28,29,30,31,32,33]. The surface microstructure of the prepared polymer had excellent controllability, a large solid−liquid contact angle and good transparency [34,35,36]. However, before constructing the surface microstructure on the template by chemical etching, it was necessary to use ultraprecision machining to produce the chemically etched template. Preparing templates by chemical etching came with some shortcomings, such as a complex preparation process and high cost. Moreover, ultrafast laser etching had also been proposed as a method to prepare superhydrophobic templates [37,38,39,40]. The prepared polymer film had good superhydrophobicity, transparency, and corrosion resistance. However, the surface microstructures etched by ultra-fast laser were mostly single-stage microstructures. The prepared templates had problems with insufficient wear resistance and short service life [41]. The reported templates for superhydrophobic polymer films have some shortcomings, such as complex preparation processes, low wear resistance, and poor reusability.

Polydimethylsiloxane (PDMS) is a kind of polymer elastomer. The low surface energy of the material makes it easy for this material to form a hydrophobic self-protective coating. Often used in the production of fiber or antipollution products, not does not pollute the environment. Moreover, PDMS coating has excellent properties such as corrosion resistance, abrasion resistance, and self-healing ability. Li prepared a superhydrophobic coating with good superhydrophobicity by spraying a fluorine-free suspension consisting of epoxy resin (EP), polydimethylsiloxane (PDMS), and modified SiO_2_ on various substrates [42]. Film prepared by the spraying method is subject to uneven film thickness and easily damaged. Using stainless-steel mesh as template, Kim prepared superhydrophobic, flexible, and gas-permeable PDMS films by a simple one-step process [43]. However, with stainless steel as the template, demolding is difficult, making it difficult to prepare a film with a complete and uniform surface texture. Toma reported the preparation of nano-conical gold films on Teflon films with nanostructures by gold-vapor deposition using PS spherical monolayer films as etching templates [44]. This method has high requirements for manufacturing equipment, comes with high costs, and is difficult to adapt to the needs of large-area preparation. The mechanical strength of the prepared superhydrophobic polymer film is not sufficient for engineering applications, so the potential for large-scale industrial production is limited.

In this paper, the existing methods “coating film” and “growing film” are replaced by an “adhesive film” method, and a method based on EDM for the single-step preparation of a multi-layer durable template is proposed; this method results in the efficient preparation of superhydrophobic polymer films. To obtain a wear-resistant and reusable template for superhydrophobic polymer films, a new preparation method is proposed based on WEDM and the template is made of 6061 aluminum alloy. Wettability and wear experiments on the template show that the contact angle of the template is greater than 150°, that it has excellent wear resistance, and that it can be used repeatedly. PDMS superhydrophobic polymer films were prepared by the template method. The properties of superhydrophobic polymer films were characterized to assess its service performance, including self-cleaning, anti-icing properties, wear resistance, self-restoration, bending resistance, and ductility. The test results show that the PDMS superhydrophobic polymer film has excellent self-cleaning, anti-icing, anti-bending, and anti-wear properties and has some self-healing properties and ductility.

## 2. Experiment and Test

### 2.1. Experiment

(1) Experimental material

The substrate of the superhydrophobic template in the experiment was 6061 aluminum alloy. This material, 6061 aluminum alloy, is a high-quality aluminum-alloy product produced through heat treatment and a prestretching process, with good formability, weldability, machinability, and moderate strength. The substrate of the polymer films in the experiment is polydimethylsiloxane (PDMS) (Dow Corning, Midland, MI, USA). PDMS consists of two components, prepolymer A and curing agent B. The composition of A is mainly poly (DimethylVinylsiloxane) prepolymer and a trace platinum catalyst. B is composed of a vinyl side chain prepolymer and a curing agent, poly (dimethyl-methylhydrogenosiloxane). When the two are mixed, the vinyl can undergo hydrogenated silanization with the hydrogen silicon bond, thus forming a three-dimensional network structure. PDMS is a polymer elastomer with low surface energy. It does not pollute the environment and can be used to produce fibers and improve antifouling and antiscaling properties.

(2) The machine tool used for WEDM

The machine tool used in the WEDM experiment was an AU300i low-speed WEDM (China, Taiwan) produced by Accutex. This machine tool is mainly composed of the numerical-control system, motion-control platform, electrode wire-threading system, discharge system, working fluid-circulation system, etc. In the experiment, the electrode wire was made of brass wire with a diameter of 0.25 mm, and the working solution was deionized water. The machining parameters used for WEDM are shown in Table 1.

(3) The preparation of superhydrophobic templates

**Figure 1:**
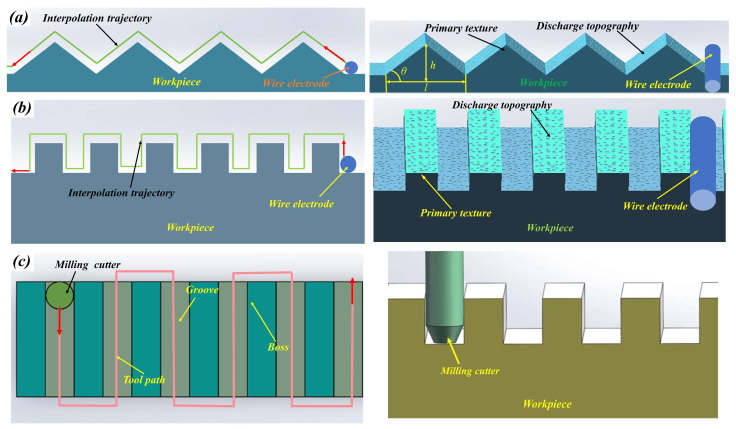
Experimental flowchart showing the preparation of superhydrophobic templates. (**a**) Triangular surface textures produced by WEDM. (**b**) Rectangular surface textures produced by WEDM. (**c**) Rectangular surface textures produced by milling.

The superhydrophobic templates were made by WEDM and milling, as shown in Figure 1. In the process of WEDM, the machining of the primary surface texture mainly relied on the interpolation trajectory of the wire electrode. The primary surface texture included triangular and rectangular surface textures. The interpolation trajectory of the wire electrode was the same as that of the primary surface texture. Due to the discharge gap, a certain bias compensation needed to be set. The secondary microstructure was mainly composed of the discharge topography from WEDM machining. In the process of WEDM, as the distance between the workpiece and the electrode decreased, a high-intensity electric field formed and broke down the dielectric between the workpiece and the electrode. Under the acceleration of the electric field, electrons on the electrode bombarded the surface of the workpiece at a high speed. Then, the material on the workpiece surface eroded and formed discharge craters. The size of a single discharge crater was very small, with a diameter of 5–20 μm. WEDM is a high-frequency pulse discharge etching process that generates 500 to 2000 discharge craters per second. Due to the randomness of the distribution of discharge points, micro/submicro discharge topography formed on the surface of the workpiece. This is the fundamental condition for preparing superhydrophobic surface hierarchical microstructures.

(4) The preparation of superhydrophobic polymer films by the template method

**Figure 2:**
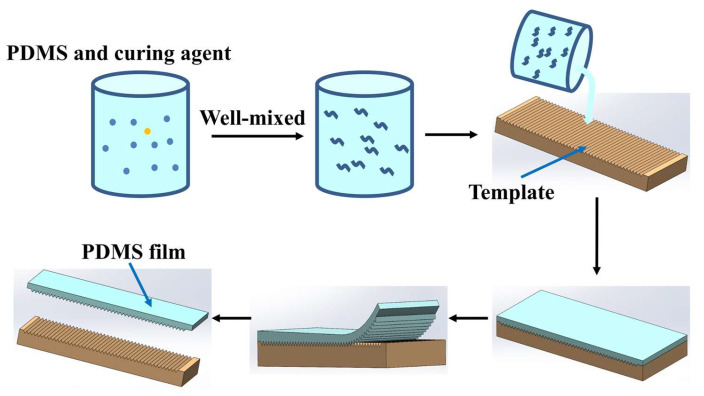
Experimental flowchart showing the preparation of superhydrophobic polymer by template method.

An experimental flowchart showing the preparation of superhydrophobic polymer by the template method is given in Figure 2. The preparation process was as follows. First, specific proportions of PDMS and curing agent were poured into a beaker. The ratio of PDMS to curing agent in this experiment was 10:1. PDMS and curing agent were thoroughly stirred and mixed. The mixed solution was allowed to stand for a period of time until all the bubbles disappeared. The superhydrophobic template was placed at the bottom of the mold. The mixed solution was evenly spread on a superhydrophobic template. The mixed solution and template were stored in a 100 °C high-temperature box for 0.5 h. After cooling, the polymer film could be separated from the template.

### 2.2. Test

(1) Surface roughness and topography

The surface roughness was measured by surface profilometer (Brook/GTK). The micro/submicro discharge topography was observed using a scanning electron microscope (MIRA 3 LMU).

(2) The size of the surface texture

The actual size of the surface texture was measured using a depth-of-field optical microscope (VHX-500, Keyence, Osaka, Japan) with a magnification of 50×.

(3) Solid−liquid contact angle

The solid−liquid contact angle on the workpiece surface was measured by a high-temperature contact-angle-measuring instrument (Theta, Biolin, Espoo, Finland). The experimental steps were as follows: 4 μL of water droplets was taken using a straw and dropped onto the surface texture of the workpiece. The dynamics of the water droplets on the surface texture of the workpiece were recorded for 10 s by the instrument’s built-in camera. The stable measurement diagrams of the contact angle were selected and recorded. The experiment was repeated 3–5 times at different positions on the same texture, and the average value was taken as the final contact-angle value.

(4) The self-cleaning performance of polymer film

The same amount of fine sand was spread evenly on the surfaces of different samples. All samples were tilted at the same angle. The same amount of water was poured onto the different sample surfaces. The flow of water and the residue of fine sand on the different sample surfaces were observed and analyzed.

(5) The anti-icing performance of polymer film

The sample was placed in a low-temperature test chamber. Then, 4 μL water droplets was dropped onto the surface texture of the sample. The entire process of the water droplets freezing was recorded by a camera. The freezing speed and dynamics of the water droplets on the surface of the workpiece were observed and analyzed.

(6) The wear resistance of template and polymer film

**Figure 3:**
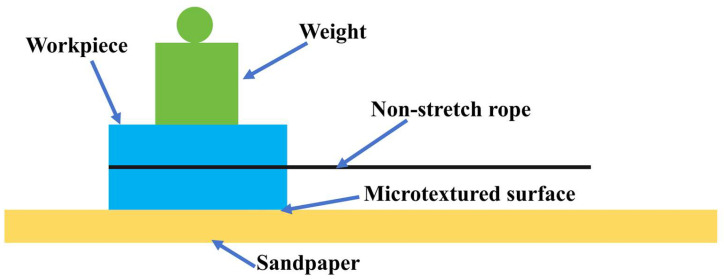
Schematic diagram of wear resistance test.

A schematic diagram of the wear-resistance test for the template and polymer film is shown in Figure 3. Sandpaper, samples, and weights were placed on the table in sequence. The rough surface of sandpaper contacted the surface microstructure of the sample. The sample was dragged and moved by a non-elastic rope. The surface microstructure of the sample became worn due to the friction between the sandpaper and the sample. In the wear test of the metal template, 400# sandpaper and 50 g weights were used. In the wear test of the polymer film, 1200# sandpaper and 20 g weights were used. The sliding distance of the weight for each cycle was 50 mm. After every 50 wear cycles, the contact angle on the sample surface was measured. On the same sample, the surface contact angles were measured at 3–5 different positions, and their average value was taken as the contact angle of the sample.

(7) The wear resistance of the template and polymer film

The template was used to repeatedly prepare superhydrophobic polymer films. The contact angle on the surface of the polymer film was measured every 10 repetitions of preparation. This method was used to evaluate the reusability of the template.

(8) The bending resistance of the polymer film

**Figure 4:**
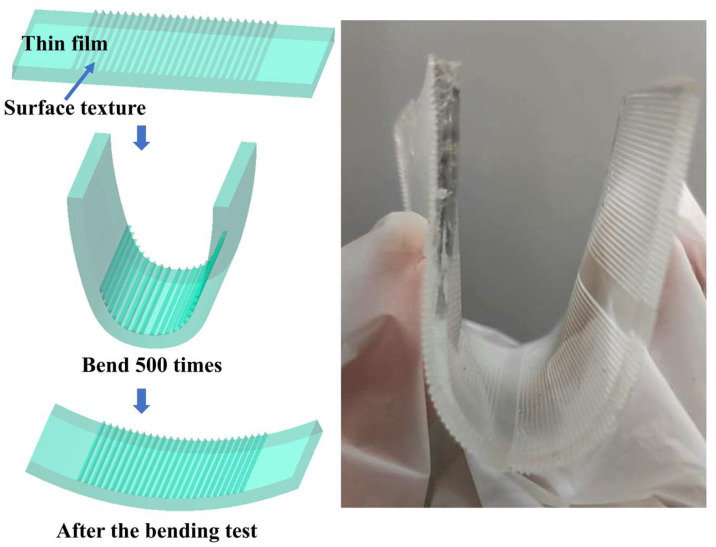
Schematic diagram of bending resistance.

The schematic diagram of the bending resistance of polymer film is shown in Figure 4. According to the diagram, the polymer film was bent 500 times. The formation of any cracks, wrinkles, or other types of damage on the film surface was observed. The surface contact angle at the bend of the polymer film was measured to evaluate the bending resistance of superhydrophobic polymer films.

(9) The ductility of polymer film

The polymer film was subjected to a tensile test to evaluate its ductility. The equipment used for the tensile test was the Instron 5943 electronic universal testing machine. The tensile strength and elongation of polymer film were measured. The surface contact angle near the tensile fracture of the polymer film was measured.

(10) The self-restoring properties of polymer film

**Figure 5:**
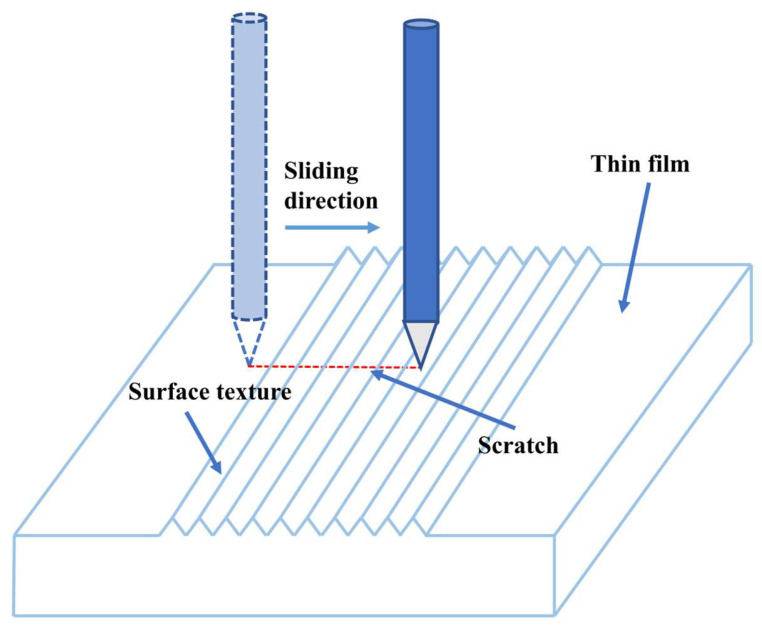
Schematic diagram of self-restoration test.

A scratch was made on the surface of the polymer film using a needle (see Figure 5). The surface contact angles of the polymer film at 10 min, 20 min, 30 min, 1 h, and 2 h after scratching were measured to evaluate its self-healing performance.

## 3. The Simulation and Analysis of Solid−Liquid Contact Angle

### 3.1. The Size Design of Template

The primary surface textures of the template in this study mainly included triangular and rectangular surface textures. The triangle surface texture was an isosceles triangle, whose design parameters included bottom edge and height. The design parameters of the rectangular surface texture included groove width, groove depth, and ridge width. The design parameters of the rectangular surface texture were as shown in Table 2. The triangle surface texture of each size consisted of three samples created by WEDM rough machining, WEDM single-pass trim machining, and WEDM double-pass trim machining. The rectangular surface texture of each size consisted of four samples, which were created by WEDM rough machining, WEDM single-pass trim machining, WEDM double-pass trim machining, and milling.

### 3.2. The Simulation Model of Solid−Liquid Contact Angle

The finite-element calculation method was applied to simulate the morphological changes of water droplets in contact with metal templates. Because the simulation was intended to simulate the contact angle of the metal template, the metal surface energy was set to be equal to the surface energy of the aluminum alloy during the simulation, and the surface energy of the metal template was 37.9 mN/m, as calculated by the two-liquid method. In the simulation process, the solid phase, liquid phase, and gas phase were involved. A total of 4 μL water droplets was released near the template surface. After the water droplets contacted the template surface, they underwent morphological changes such as rebound, fall, and spread. After a period of time, the water droplets became stationary on the template surface. Then, the static solid−liquid contact angle could be measured. The simulation parameters were as follows. The ambient temperature was set to 200 °C. The surface tension of the liquid phase (water) in the air interface (i) was 7.275 × 10^−2^ N/m. The density of water (ρ) was 998.2 kg/m^3^. The viscosity of the water (μ_1_) was 1.002 × 10^−3^ Pa·s. The gravitational acceleration of water (g) was 9.8 m^2^/s. The density of the gas phase (air) (ρ) was 1.205 kg/m^3^. The air viscosity (μ_2_) was 2.593 × 10^−5^ Pa·s. The contact angle of the solid phase wall was set as the apparent contact angle of the specimen created by WEDM, as obtained from the experiment.

Based on the size design of the surface texture and the simulation model of the contact angle, the contact topography of template and water droplets on the surface texture could be obtained, as shown in Table 3. The solid−liquid contact angle could be obtained by measuring the shape of the water droplets using JC2000G software. From the simulation results, the following points could be seen. (1) The solid−liquid contact angle on some surface textures exceeded 150°. The maximum solid−liquid contact angle was 155.3°, which reflected a superhydrophobic state. The solid−liquid contact state was in Cassie−Baxter form. (2) The solid−liquid contact angle on the triangular surface texture was generally bigger than that on the rectangular surface texture. (3) The contact angle of the template surface decreased with the increase in surface-texture gap and decreases with the decrease in surface-texture depth. When the depth of surface texture was below a certain value, water droplets fell to the surface of the template and touched the bottom of the surface texture due to gravitational action. The solid−liquid contact state then transformed from Cassie−Baxter topography to Wenzel topography, and the solid−liquid contact angle also decreased. According to the simulation results, in order to obtain a large surface contact angle on polymer film, the design dimensions given as No.2, No.3, No.6 and No.7 in Table 2 were selected as the preparation dimensions for the triangular texture template and were respectively recorded as size I, size II, size III, and size IV. The design dimensions given as No.3, No.4, No.11, and No.16 in Table 2 were selected as the preparation dimensions for the rectangular texture template and then respectively recorded as size I, size II, size III and size IV.

## 4. Results and Discussion

### 4.1. Template

(1) Surface Roughness and Topography

**Figure 6:**
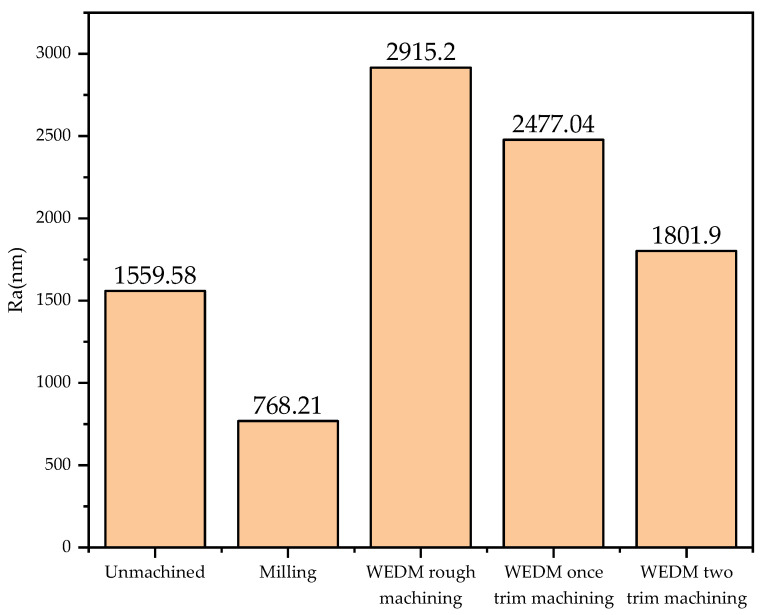
Surface roughness of workpieces processed using different technologies.

The optical profilometer was used to observe and measure the surface roughness of the aluminum alloy obtained by different machining methods. The results are shown in Figure 6. Compared with the surface of the unprocessed aluminum alloy, the surface roughness of the aluminum-alloy plate processed by EDM wire cutting was higher, and that of the workpiece roughed by EDM wire cutting was the highest. The roughness of the workpiece was reduced after the trimming treatment, but it was still higher than that of the unprocessed aluminum-alloy plate. By contrast, the surface roughness of the milled aluminum alloy was reduced compared to that of the unprocessed aluminum-alloy plate.

(2) Surface topography

**Figure 7:**
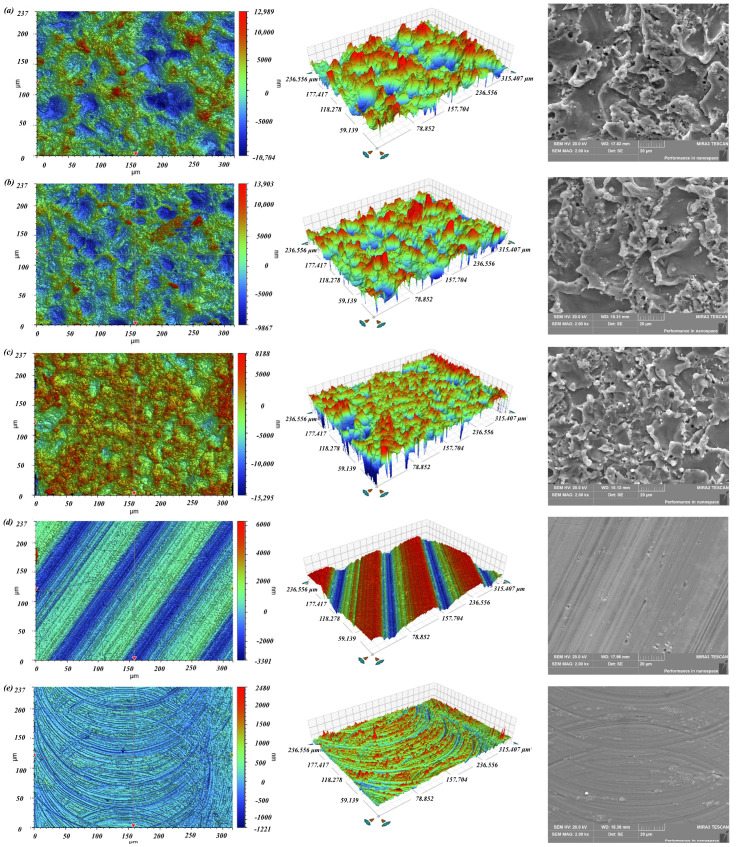
The surface topography of different specimens, as determined by surface profilometer and SEM. (**a**) WEDM rough machining. (**b**) WEDM single-pass trim machining. (**c**) WEDM double-pass trim machining. (**d**) Milling (ridge). (**e**) Milling (groove).

The surface roughness of different specimen is measured by surface profilometer. The surface topography of different specimen is measured by surface profilometer and SEM, as shown in Figure 7. The surface roughness of an unmachined surface and specimens produced by milling, WEDM rough machining, WEDM single-pass trim machining and WEDM double-pass trim machining were 1.56 μm, 0.77 μm, 2.92 μm, 2.48 μm and 1.80 μm, respectively. From the above results, it can be seen that the surface roughness of samples produced by milling was the lowest. The surface roughness of samples produced by WEDM was higher than that of the unprocessed surface. This is mainly because the micro/submicro discharge topography was constructed on the specimen surface. In addition, as the number of trim machining increased, the surface roughness gradually decreased. This is because the discharge energy of a single pulse in precision machining was lower, the feed rate was smaller, and the erosion of the dielectric was more thorough, resulting in a smaller size of discharge craters and easier removal of discharge erosion residues. Moreover, it can be also found that the micro/submicro discharge topography mainly included pits and protrusions. The size of these pits and protrusions was about 0.5–30 μm. As the surface roughness of the sample decreased, the size of pits and protrusions gradually decreased.

(3) Solid−liquid contact angle

**Figure 8:**
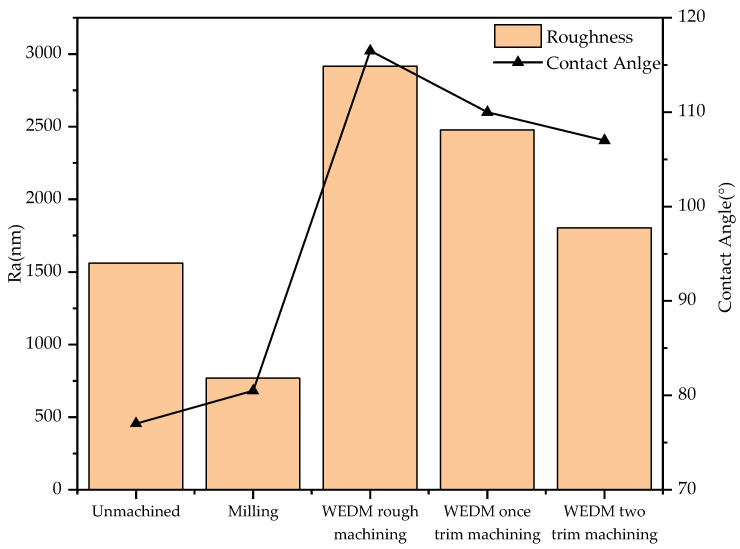
Graph showing the relationship between roughness and contact angle.

The apparent contact angle of different specimens was measured by a high-temperature contact-angle-measuring instrument. The apparent contact angles of the unmachined surface and samples produced by milling, WEDM rough machining, WEDM single-pass trim machining and WEDM double-pass trim machining were 77°, 80.5°, 116.5°, 110°, and 107°, respectively. From this, it can be seen that the apparent contact angle of the samples processed by WEDM was significantly greater than that of the untreated sample surface and the milled sample surface. This is mainly because the micro/submicro discharge topography that formed after WEDM was conducive to the formation of an air-cushion effect during the solid−liquid contact process, reducing the solid−liquid contact area and thereby increasing the solid−liquid contact angle. In addition, the apparent contact angle of the sample after WEDM rough machining was the largest, while the apparent contact angle of the sample after WEDM trim machining was slightly reduced, as shown in Figure 8. It can be concluded that with the reduction in roughness, the contact angle decreased, that there was a positive correlation between the contact angle and the roughness in a certain range, and that the larger discharge morphology was conducive to promoting the air-cushion effect and increasing the apparent contact angle. Although the roughness of the surface after milling was lower than that of the unmachined surface, during milling, some fine structures on the tool created microstructures on the surface of the aluminum alloy, resulting in an increase in the contact angle.

According to Figure 8, it can be seen that the contact angle on the specimen surface machined by WEDM rough machining was the highest. Therefore, WEDM rough machining was adopted for processing surface texture. The solid−liquid contact angle on the surface texture of different templates was measured by high-temperature contact angle measuring instrument, as shown in Table 4. From Table 4, it can be seen that the maximum contact angle on the triangular surface texture and the rectangular surface texture exceeds 150°, which reach superhydrophobic state. Overall, the contact angle on the triangular surface texture was higher than that on the rectangular surface texture. In the triangular surface texture, when the ratio of height to bottom was small, water droplets tended to collapse and come into contact with the bottom of the surface texture, resulting in a smaller contact angle. The contact angle on the rectangular surface texture processed by WEDM was significantly greater than that of the rectangular surface texture processed by milling. This is mainly because the micro/submicro discharge topography was formed on the surface texture after WEDM rough machining, which was conducive to the formation of air-cushion effect and thus reduces the solid−liquid contact area.

**Figure 9:**
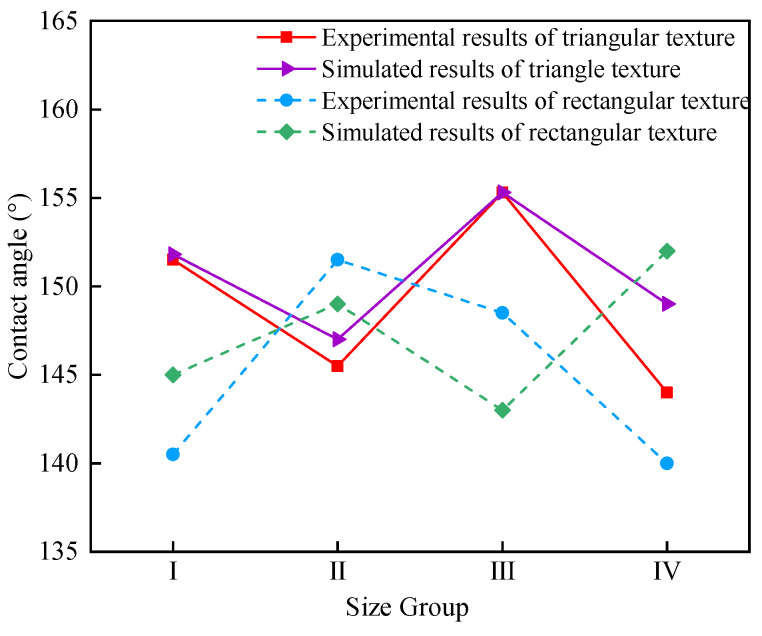
The comparison between experimental and simulated values of contact angle.

Figure 9 shows the comparison between experimental and simulated values of contact angle. The simulation value of the solid−liquid contact angle was in good agreement with the experimental value, and the average error was 1.14%. From this, it can be seen that the established solid−liquid contact angle simulation model has high accuracy, which can guide the design of superhydrophobic templates.

(4) Wear resistance

**Figure 10:**
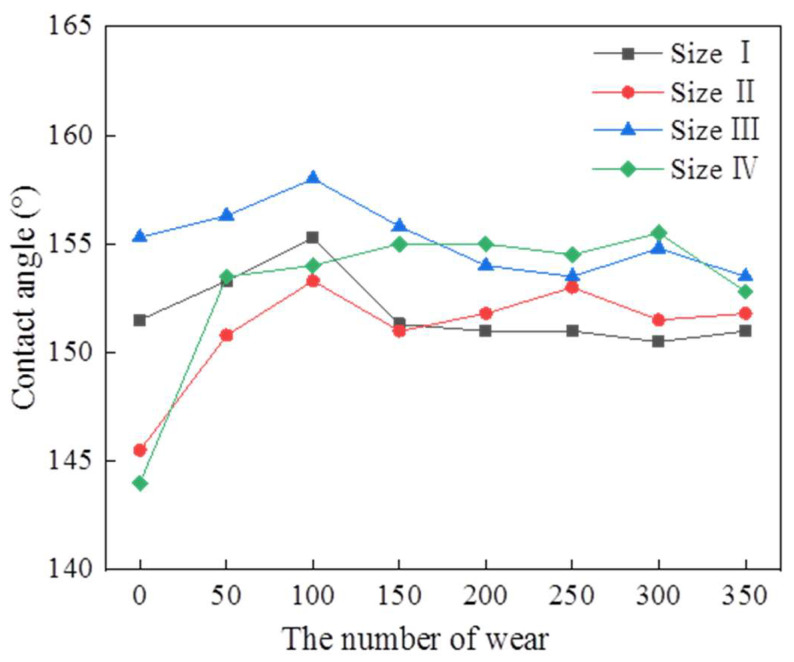
The surface contact angle on triangular texture template after different times wear.

Figure 10 shows the surface contact angle on the triangular texture template after different wear cycles. It can be seen that there was no obvious decrease in the surface contact angle on the template after 350 wear cycles. This is mainly because the triangular texture on the template surface becomes trapezoidal in texture after sandpaper wear. The discharge topography at the top of the triangular texture will be destroyed. However, the discharge topography on the side of the triangular texture cannot be damaged. Together with the trapezoidal texture, the discharge topography can still form an air-cushion support to reduce the solid−liquid contact area. Hence, it can be said that the template with triangular surface texture prepared by WEDM has good wear resistance.

### 4.2. Polymer Film

(1) Demolding residue

**Figure 11:**
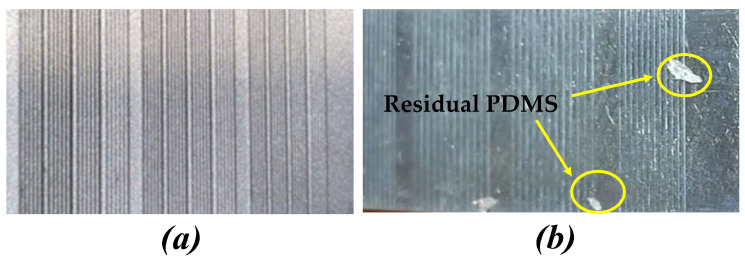
The residue on the template after demolding. (**a**) Triangular surface texture. (**b**) Rectangular surface texture.

According to the above analysis, the contact angle on triangular surface texture is larger than that on rectangular surface texture. In addition, as shown in Figure 11, it can be observed that during the demolding process of polymer films, there were very few residues on the triangular surface texture template and that the residues were almost invisible to the human eye. The surface texture of the prepared polymer film was complete and defect-free. However, there were many PDMS residues on the rectangular surface texture template. The surface-texture defects of the prepared polymer film were obvious, and there were many pit defects. Therefore, choosing the triangular surface texture as the final template is more appropriate.

(2) Molding rate

**Figure 12:**
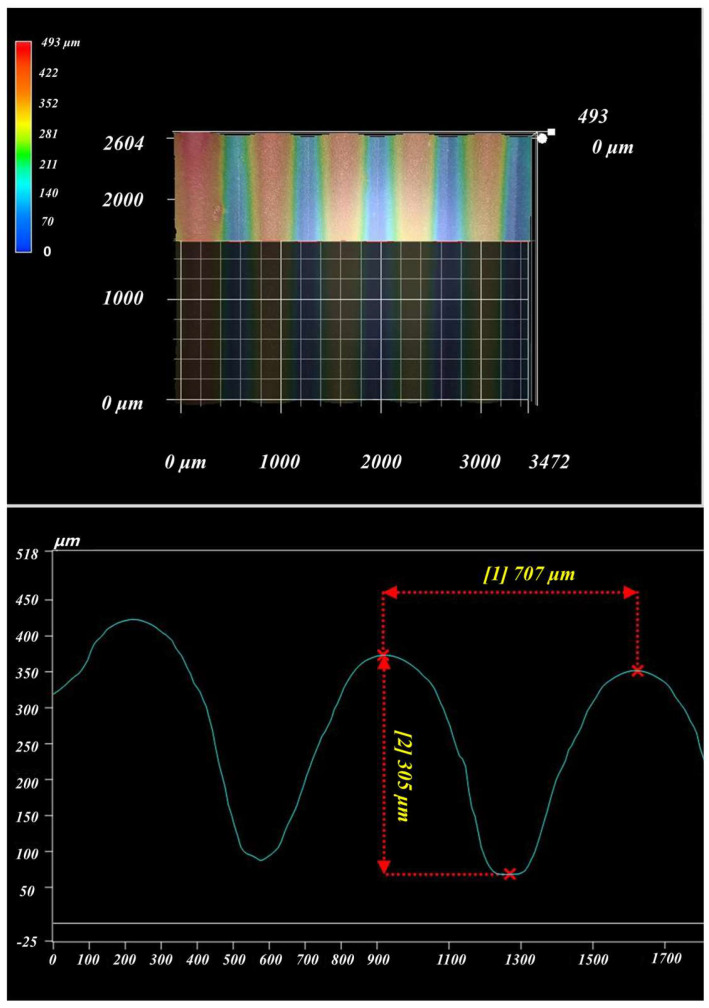
The measurement results of surface texture profile.

Figure 12 shows the measurement results of the surface texture on polymer films by a super depth of field microscope. From Figure 12, it can be seen that the shape of the surface texture was basically the same. The height and width of different contours were generally the same. Table 5 shows the size comparison of triangle surface-texture template and polymer film. It was found that the surface texture size on the polymer film was very close to that on the template, with a relative error of less than 3%. This means that polymer films prepared using triangular surface-texture templates have a higher molding rate.

(3) Solid−liquid contact angle

**Figure 13:**
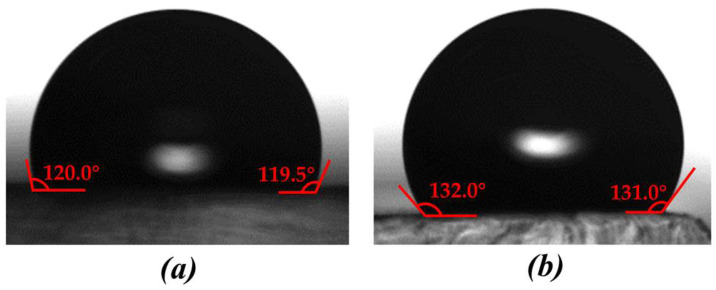
The measured result of contact angle on polymer films without surface texture. (**a**) Unprocessed surface, 119.8°. (**b**) WEDM surface, 131.5°.

**Figure 14:**
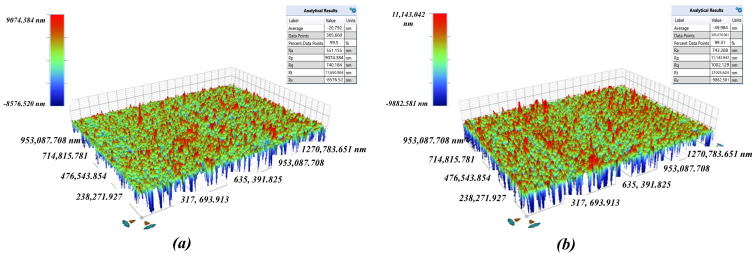
Surface roughness of PDMS films. (**a**) Unprocessed surface, Ra 551 nm. (**b**) WEDM surface, Ra 743 nm.

**Figure 15:**
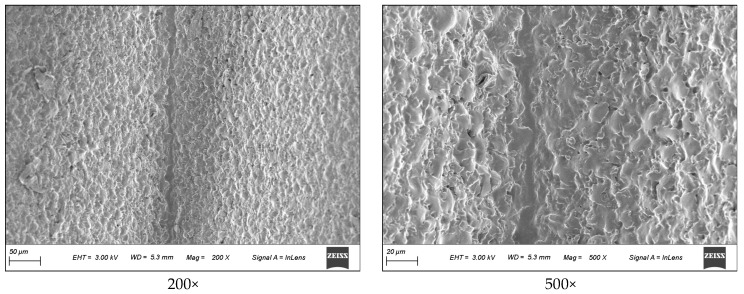

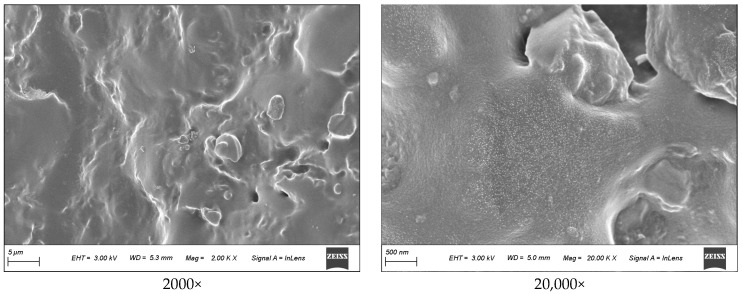
The SEM photograph of the thin film surface.

**Figure 16:**
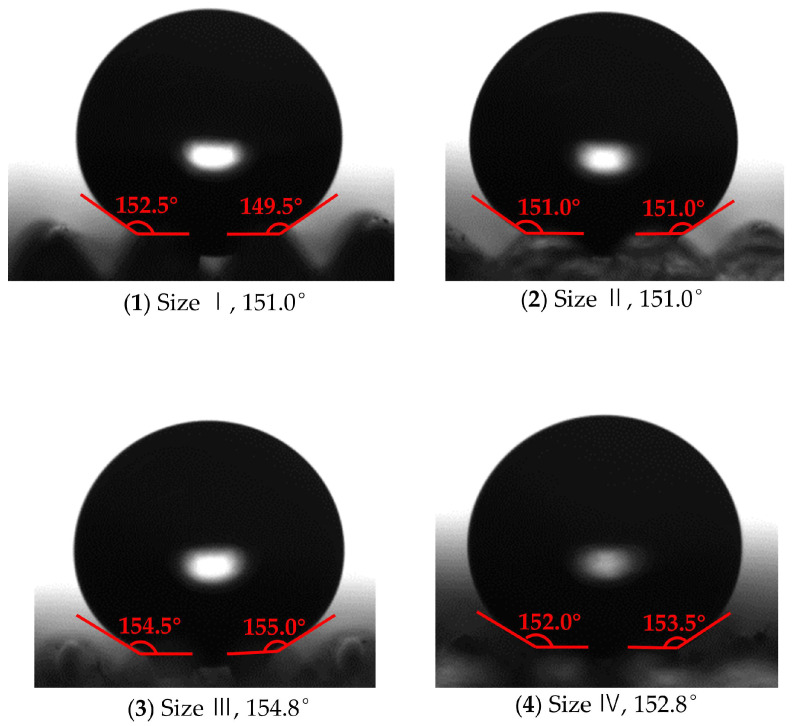
The measured results for contact angle on polymer films with triangular surface texture.

Figure 13 shows the measured result of contact angle on polymer films without surface texture. The templates that generated the results shown in Figure 13a,b were an unprocessed surface and a WEDM surface, respectively. The contact angles on Figure 13a,b were 119.8° and 131.5°, respectively. This is mainly due to the fact that, although there was no surface texture, the micro/submicro discharge topography of WEDM can be replicated onto the surface of polymer films, where it can enhance the air-cushion effect and reduce the solid−liquid contact angle. The surface energies of the films were calculated by the two-liquid method [45], and the surface energy of the film was reduced from 22.156 mN/m to 18.632 mN/m. The roughness of the two films was measured using an optical profiler, and the results showed that the roughness of the films prepared by the EDM template was higher than that of the unmachined films (Figure 14), which indicates that higher roughness is conducive to forming a higher contact angle. Figure 15 shows the SEM image of the film, and it can be seen that there were many pits and bumps on it. This is because after EDM machining, due to the discharge of electrode wire, pits and bumps formed on the surface of the template and were copied to the film. Figure 16 shows the measurements of contact angles on polymer films with triangular surface texture. It can be seen that all contact angles exceeded 150°. The maximum contact angle was 154.8°. In addition, it can be observed that the solid−liquid contact states on the surfaces of Size I, Size II, and Size III were the Cassie−Baxter state. The solid−liquid contact state on the surface of Size IV was the Wenzel state. This means that the prepared polymer film attained a superhydrophobic state through the combination of a triangular surface texture and WEDM rough machining.

(4) Reusability

The same template was used to repeatedly prepare superhydrophobic polymer film. The number of repetitions was 50. The measured contact angles on the template and the polymer-film surface are shown in Table 6. Compared to Figure 16 and Table 6, after 50 repetitions of preparation, there was no significant change in the contact angle on the surface of the template and the polymer film. All contact angles on the polymer-film surface were larger than 150°. Hence, it can be said that the template with triangular surface texture prepared by WEDM has good reusability.

(5) Wear resistance

**Figure 17:**
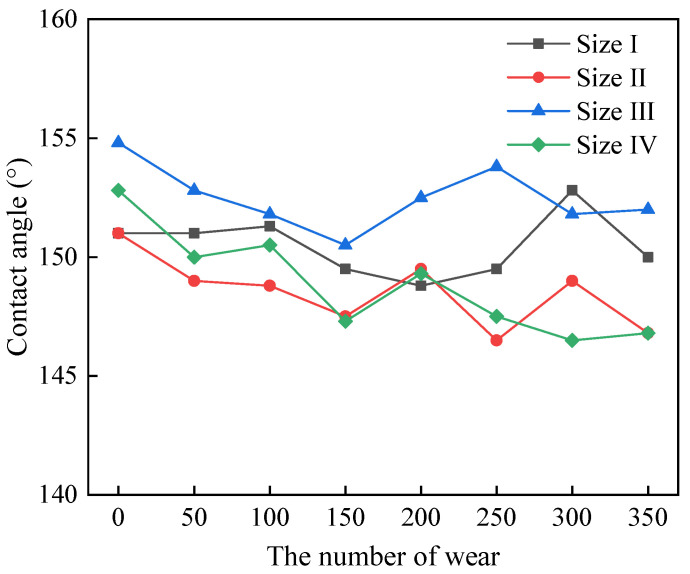
The surface contact angle on polymer film after different times wear.

Figure 17 shows the surface contact angle on polymer film after different numbers of wear cycles. It can be seen that the contact angle on the surface of the polymer film slightly decreased with wear. Besides, the contact angle on the triangular textured surfaces of size I and size III was still larger than 150°, maintaining superhydrophobic state. This is mainly because that the wear resistance of polymer material is not as good as that of metal material. The top of the surface texture on the film is prone to wear. The height of the surface texture was reduced. Water droplets on the surface of polymer films tend to contact the bottom of the surface texture. This may cause the solid−liquid contact state to transition from the Cassie−Baxter state to the Wenzel state. Therefore, it can be said that the prepared superhydrophobic polymer film has high wear resistance.

(6) Self-restoring

**Figure 18:**
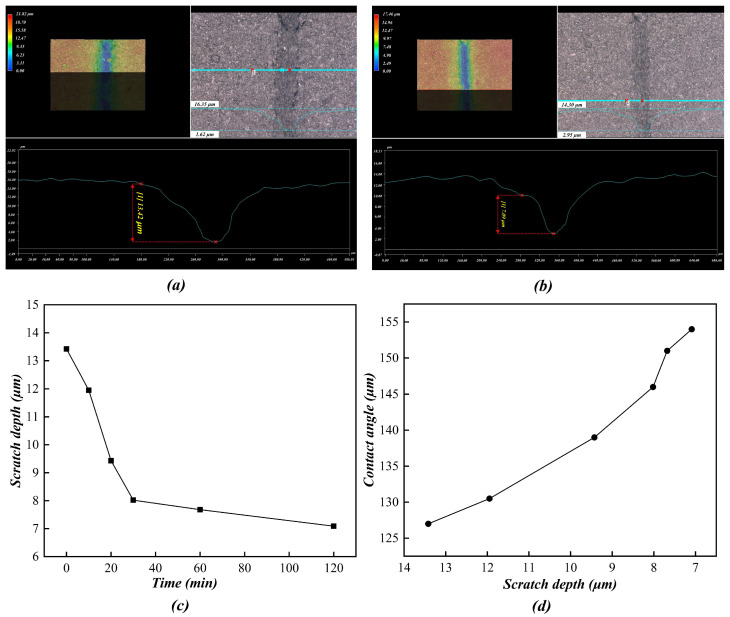
Measurements of scratches on the polymer-film surface. (**a**) 0 min, 13.42 μm. (**b**) 120 min, 7.09 μm. (**c**) The trend in scratch depth over time. (**d**) The relationship between scratch depth and contact angle.

Figure 18 shows the results from measuring scratches on the polymer-film surface. It can be seen that there was a clear scratch (depth of 13.42 μm) on the surface of the polymer film just after the film was scratched. As the standing time increased, the scratch on the surface of the polymer film gradually reduced in size and the depth of the scratch gradually became shallower. After the film had stood for 120 min, the scratch depth was reduced to 7.09 μm. After the film had stood for 24 h, the scratch was almost invisible to the human eye.

Figure 18d shows the relationship between scratch depth and contact angle. It can be observed that, just after the film was scratched, the contact angle on the polymer-film surface was only 127°. This is mainly because that the scratch damaged the hierarchical structure of the polymer-film surface. As the standing time increased, the surface contact angle on the polymer film gradually increased. After the film had stood for 120 min, the surface contact angle on the polymer film was 154° and the superhydrophobicity of the polymer film was restored. Therefore, it can be said that the prepared superhydrophobic polymer film has good self-healing capacity.

(7) Bending resistance

**Figure 19:**
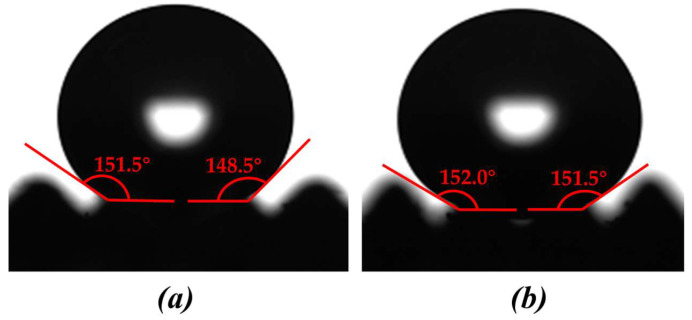
Surface contact angle on polymer film after different bending times. (**a**) 150°. (**b**) 151.8°.

Figure 19 shows the surface contact angle on polymer film after different numbers of bending cycles. It can be observed that the contact angle on the polymer film slightly decreased with the increase in the number of bending cycles. After it had been bent 500 times, there was no obvious crease, crack or fold on the surface of the polymer film. The surface contact angle on the polymer film still exceeded 150°. Therefore, it can be said that the prepared superhydrophobic polymer film has good bending resistance.

(8) Ductility

**Figure 20:**
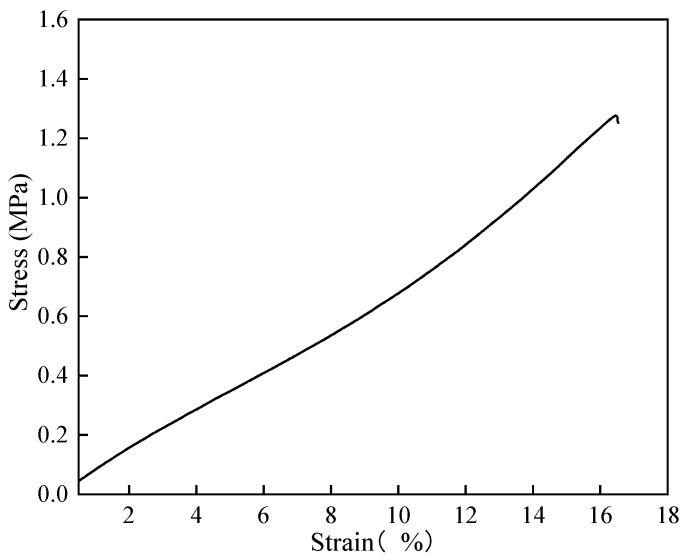
Stress-strain curve of polymer film in the tensile test.

**Figure 21:**
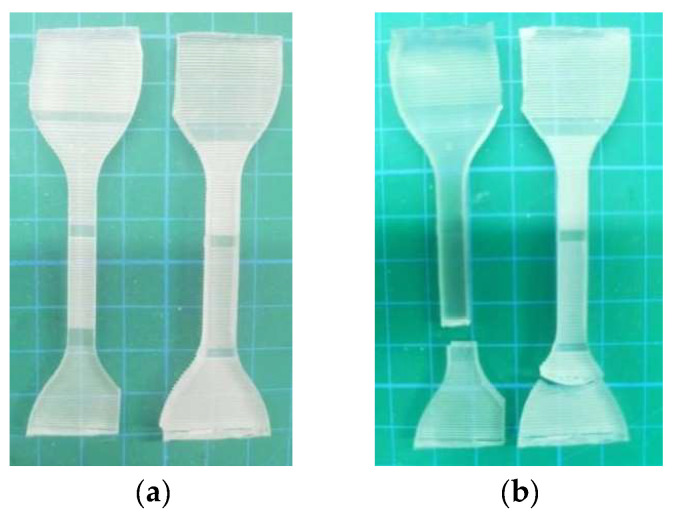
Polymer film before and after tensile test. (**a**) Before tensile test. (**b**) After tensile test.

Before the tensile test, the film thickness was measured with vernier caliper, and the average value was taken for five measurements. The film thickness was 2.04 mm. Figure 20 and Figure 21 shows the stress-strain curve of polymer film in the tensile test. It can be seen that, unlike metal materials, polymer film will immediately fracture when the stress exceeds the critical value. The maximum stress that the polymer film could withstand was 1.27 MPa, and the elongation was 16.53%. After the tensile test, the surface contact angle near the fracture of the polymer film exceeded 150°, at 151.6° and 156°. That is, polymer film after tensile testing still exhibited superhydrophobicity. Therefore, it can be said that the prepared superhydrophobic film has excellent ductility and tensile strength.

(9) Self-cleaning

**Figure 22:**
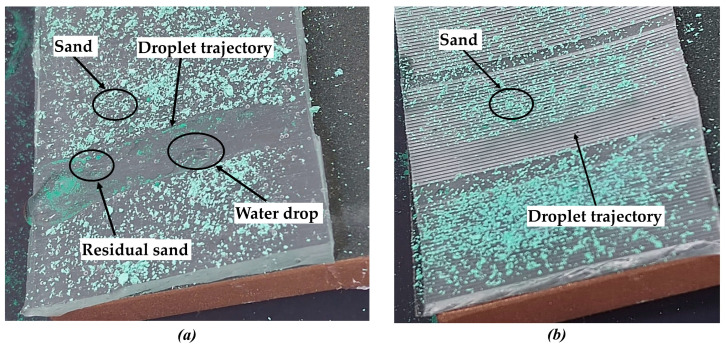
The result of the self-cleaning experiment on different polymer films. (**a**) Polymer films without surface texture. (**b**) Polymer films with triangular surface texture.

Figure 22 shows the result of the self-cleaning experiment on different polymer films. As shown in Figure 22a, after the water droplets had been dropped onto the polymer film, the surface fine sand could not be completely removed with the water droplets. Some of the fine sand mixed with the water and remained on the surface of the polymer film. As shown in Figure 22b, after the water droplets had fallen on the polymer film, water droplets quickly rolled off the surface of the polymer in a spherical shape. The fine sand on the polymer surface was completely carried away by the water droplets in the path the water droplets passed through. This indicates that the prepared polymer film has good self-cleaning performance.

(10) Anti-icing

**Figure 23:**
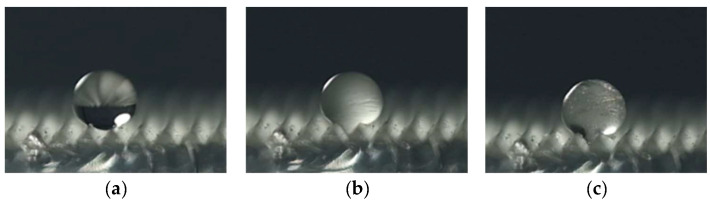
Images of the water-droplet freezing process (Size I). (**a**) Not frozen. (**b**) Start of freezing. (**c**) Full freezing.

A drop of water (4 μL) was dropped onto polymer film and placed in a low-temperature test chamber at a temperature of −12 °C. The camera was applied to observe and record the entire process of the water droplet freezing as shown in Figure 23. Table 7 shows the freezing schedule of water droplets on different polymer-film surfaces. Comparing the template of the unprocessed surface with the template created by WEDM without surface texture, it can be seen that the micro/submicro discharge topography of WEDM can delay the start of time to freezing but has little effect on the overall time to freezing. This is mainly due to the fact that the single-stage surface microstructure can reduce the solid−liquid contact area and heat-transfer area through the air-cushion effect. Thus, the start time of freezing is delayed. However, as the icing process progresses, the water droplet volume expands and the air-cushion effect of the single-stage surface microstructure is destroyed. The heat-transfer area is the same as that of a regular surface and cannot increase the overall time to freezing. Moreover, comparing the template of Size I with the template created by WEDM without surface texture, it can be seen that the multi-level hierarchical microstructure can obviously increase the start time and overall time of freezing. The start time and overall time of freezing were 892 s and 970 s, respectively. This is mainly because, under the combined effect of the primary surface texture and secondary discharge topography, the contact state between water droplets and polymer films was the Cassie−Baxter state, which can significantly reduce the solid−liquid contact area. In addition, the air layer between the water droplets and the polymer film can reduce the heat-transfer rate. Furthermore, comparing the template of Size I with Size IV, it can be observed that the anti-icing ability of the surface in the Cassie−Baxter state was significantly better than that of the surface in the Wenzel state. This is mainly because there is no air layer between water droplets and the surface of a polymer film in the Wenzel state.

## 5. Conclusions

(1) In terms of superhydrophobicity: Given the discharge topography created by WEDM rough machining and the appropriate triangular surface texture, the maximum solid−liquid contact angle on the aluminum-alloy template can reach up to 155.3°. The maximum solid−liquid contact angle of polymer film is 154.8°.
(2) In terms of the wear resistance and reusability of the polymer template: The multi-level hierarchical microstructure of aluminum-alloy templates has high wear resistance to 400# sandpaper with a 50 g weight mass. It has been proven that the contact angle of polymer films prepared using polymer templates remains basically unchanged after multiple repeated uses.
(3) In terms of the mechanical strength of polymer films: the prepared polymer films had been proven to have good wear resistance, self-restoring capacity, bending resistance, and ductility.

The established solid−liquid contact-angle simulation model can be used to guide the design of surface textures. The proposed preparation method has good potential for engineering applications and improvements, as in solar cells and optical glass.

## Figures and Tables

**Table 1:** The machining parameters used for WEDM.

Machining Parameters		Rough Cutting	Trim Cutting
Open-circuit voltage	V	35	35
Pulse on time	ns	300	150
Pulse off time	μs	15	8
Arc on time	ns	250	150
Arc off time	μs	7	8
Servo voltage	V	22	40
Wire tension	g	1200	1400
Cutting speed	mm^2^/min	11	7

**Table 2:** The design parameters of rectangular surface texture (μm).

No.	Triangular Texture		Rectangular Texture		
	Edge Length	Height	Ridge Width	Groove Width	Groove Depth
1	800	400	300	300	200
2	800	350	300	300	250
3	800	250	300	300	350
4	800	210	300	300	400
5	700	350	300	400	200
6	700	300	300	400	230
7	700	220	300	400	250
8	700	150	300	400	300
9	600	280	300	400	350
10	600	250	300	400	400
11	600	200	400	300	300
12	600	150	400	300	350
13	500	230	400	300	400
14	500	200	400	400	350
15	500	180	400	400	400
16	500	150	400	400	450

**Table 3:** The simulation results for contact angle.

No.	Contact Angle (°)	
	Triangular Texture	Rectangular Texture
1	139.0	117.5
2	151.8	138.5
3	147.0	149.0
4	139.5	152.0
5	135.8	126.5
6	155.3	132.5
7	149.0	135.8
8	135.8	137.0
9	118.3	138.5
10	124.5	139.3
11	119.8	145.0
12	118.0	133.5
13	119.0	135.3
14	117.5	138.5
15	120.5	132.5
16	117.8	143.0

**Table 4:** The solid−liquid contact angle on the surface texture of different templates.

No	Triangular Surface Texture by WEDM Rough Machining (°)	Rectangular Surface Texture by WEDM Rough Machining (°)	Rectangular Surface Texture by Milling (°)
Size I	151.5	140.5	126.0
Size II	145.5	151.5	145.5
Size III	155.3	148.5	140.5
Size IV	144.0	140.0	121.5

**Table 5:** The size comparison of triangle surface texture template and polymer film.

No.	Edge Length		Height	
	Template	Polymer Films	Template	Polymer Films
I	792	804	384	406.6
II	782	787	243	243.0
III	694	707	297	303.8
IV	723	710	174	170.3

**Table 6:** The contact angles of the template and film surface after repeated tests.

Template		Polymer Film	
No.	Contact Angle (°)	No.	Contact Angle (°)
Size I	156.0	Size I	150.0
Size II	149.5	Size II	150.0
Size III	151.5	Size III	154.5
Size IV	145.5	Size IV	150.5

**Table 7:** Freezing schedule of water droplets on different polymer-film surfaces.

No.	Time to Start of Freezing (s)	Overall Time to Freezing (s)
Unprocessed surface	58	256
WEDM without surface texture	99	255
Size I	892	970
Size II	463	633
Size III	612	733
Size IV	175	266

## Data Availability

The original contributions presented in the study are included in the article, further inquiries can be directed to the corresponding author.
